# Point-focusing monochromator crystal realized by hot plastic deformation of a Ge wafer

**DOI:** 10.1107/S0021889808016282

**Published:** 2008-06-14

**Authors:** Hiroshi Okuda, Kazuo Nakajima, Kozo Fujiwara, Kohei Morishita, Shojiro Ochiai

**Affiliations:** a Department of Materials Science and Engineering, Kyoto University, Japan; b Institute for Materials Research, Tohoku University, Japan

**Keywords:** Johansson monochromator, Ge, hot plastic deformation

## Abstract

Pre-polished Ge(111) single-crystal wafers were deformed just below the melting temperature to prepare point-focusing Johansson monochromator crystals. The (111) lattice plane had curvature 2*R* in the focusing plane and *R* perpendicular to it, with a hemispherical inner surface with a radius of *R* = 600 mm. By using Cu *K*α radiation, the diverging X-ray beam was focused onto a small spot.

## Abstract 2

A point-focusing Johansson monochromator crystal prepared by hot-pressing a Ge single-crystal wafer is demonstrated. By using 333 diffraction, Cu *K*α radiation was focused onto a small spot.

## 1. Introduction

Shaping monochromator crystals to realize a well focused and strong X-ray beam from a conventional X-ray generator has long been one of the most important subjects in X-ray instrumentation. Johann (1931 ▶) demonstrated a focusing curved monochromator, and Johansson (1933 ▶) proposed an advanced design of a curved crystal having the crystal surface exactly on the focusing circle. Generally, deforming perfect crystals without deteriorating their crystal quality and deforming crystals into an arbitrary shape for compact optics are not compatible. Therefore, previous work has mostly concentrated either on small and precise deformation without introducing lattice defects (Suortti *et al.*, 1986 ▶; Collart *et al.*, 2005 ▶; Stockmeier *et al.*, 2008 ▶) or on large deformation with relatively broad diffraction peaks (Allianelli *et al.*, 2004 ▶). In previous communications, we have demonstrated that large plastic deformation of semiconducting wafers such as Si and Ge is possible without severe deterioration of crystal quality (Nakajima, Fujiwara *et al.*, 2005 ▶; Okuda *et al.*, 2006 ▶). This result implies that hot plastic deformation can be used to prepare a point-focusing monochromator for applications that require moderate angular resolution and high photon flux, such as diffuse scattering and qualitative powder diffraction. In the present communication, we demonstrate that a point-focusing monochromator crystal has been prepared by a rather simple method of pre-polishing and hot plastic deformation.

## 2. Experimental

**Figure 1:**
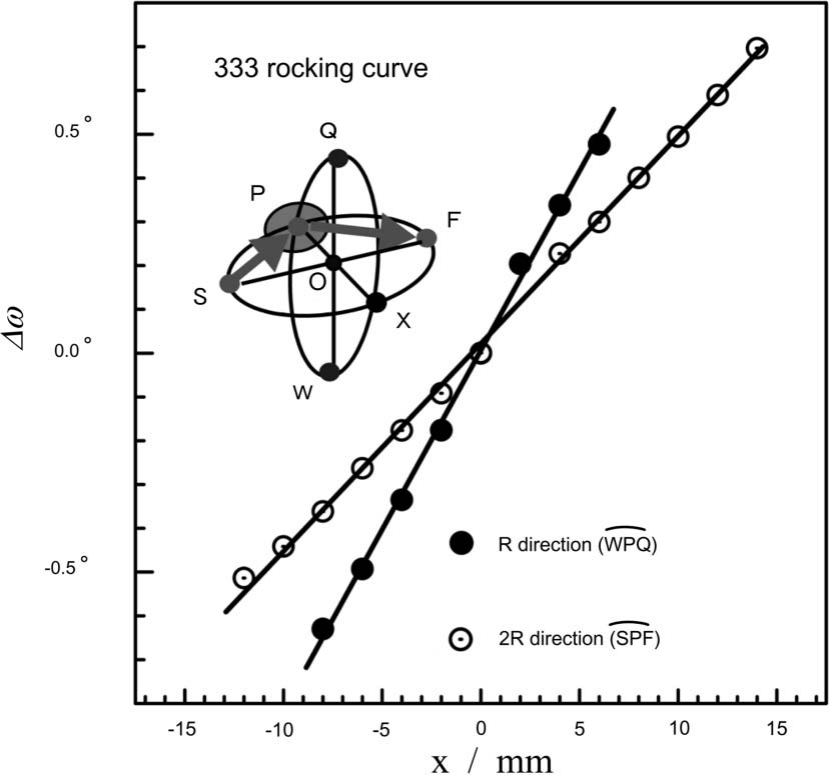
A schematic illustration of the Johansson monochromator and the curvature of the (111) plane observed by rocking scans of the crystal. The curvature is measured by the 333 peak shift of the rocking scans as a function of distance from the centre of the crystal in the *SPF* (2*R*) and *WPQ* (*R*) directions.

Ge(111) wafers 0.75 mm thick were plastically deformed at high temperatures ranging from 1173 to 1206 K in a furnace under an Ar atmosphere (Nakajima *et al.*, 2004 ▶; Nakajima, Ohdaira *et al.*, 2005 ▶; Okuda *et al.*, 2006 ▶). Ge 333 diffraction with Cu *K*α radiation gives the condition that the X-ray source point, *S*, and the focus, *F*, are at the ends of the diameter of the focusing circle. Therefore, the crystal surface of a point-focusing monochromator is spherical, as shown schematically in Fig. 1 ▶. The Ge wafers were mechanically polished to form a cylindrically concave surface with a radius of curvature of 1200 mm prior to hot deformation. They were then deformed plastically into a hemispherical shape with an inner radius of 600 mm at a temperature close to the melting point. The curvature of the (111) plane was examined by the peak shift of the 333 diffraction in an ω scan with a channel-cut incident monochromator (Okuda *et al.*, 2006 ▶).

For the focusing experiment, X-rays were generated by a Rigaku Micro7 microfocus generator operated at 40 kV and 30 mA with a Cu rotating anode.

## 3. Results and discussion

By applying a deformation at a temperature close to the melting point, we have already shown that flat Si crystals can be deformed in either a hemispherical or a cylindrical shape with a large range of radii ranging from 30 to 600 mm, while maintaining the lattice plane perpendicular to the surface (Okuda *et al.*, 2006 ▶, 2007 ▶). The realization of point-focusing monochromators satisfying Johansson’s condition is another challenge, since we must control two curvatures simultaneously, *i.e.* that of the lattice plane and that of the crystal surface. Polishing a crystal surface in a spherical or ellipsoidal shape after hot plastic deformation is not cost efficient. Therefore, we polished the crystal surface before hot deformation to give an appropriate offset angle between the surface and the lattice plane. To evaluate the deformed crystal quantitatively, rocking curves for 333 diffraction were measured using Cu *K*α_1_ characteristic X-rays monochromated by a channel-cut Ge crystal.

Fig. 1 ▶ gives the shift of the peak position in the rocking scans as a function of distance from the centre of the crystal. By moving the crystal in the in-plane directions, the peak position in the rocking scan moves, corresponding to the curvature of the (111) lattice plane. In the present case, we needed to prepare a crystal whose radius of curvature is *R* for the surface and 2*R* for the lattice plane in the *SPF* direction, and with radii for both the surface and the lattice plane of *R* in the *WPQ* direction, which is perpendicular to the *SPF* plane. The figure shows that the ratio of the two slopes of the peak shift agrees with Johansson’s condition for lattice plane curvature.

**Figure 2:**
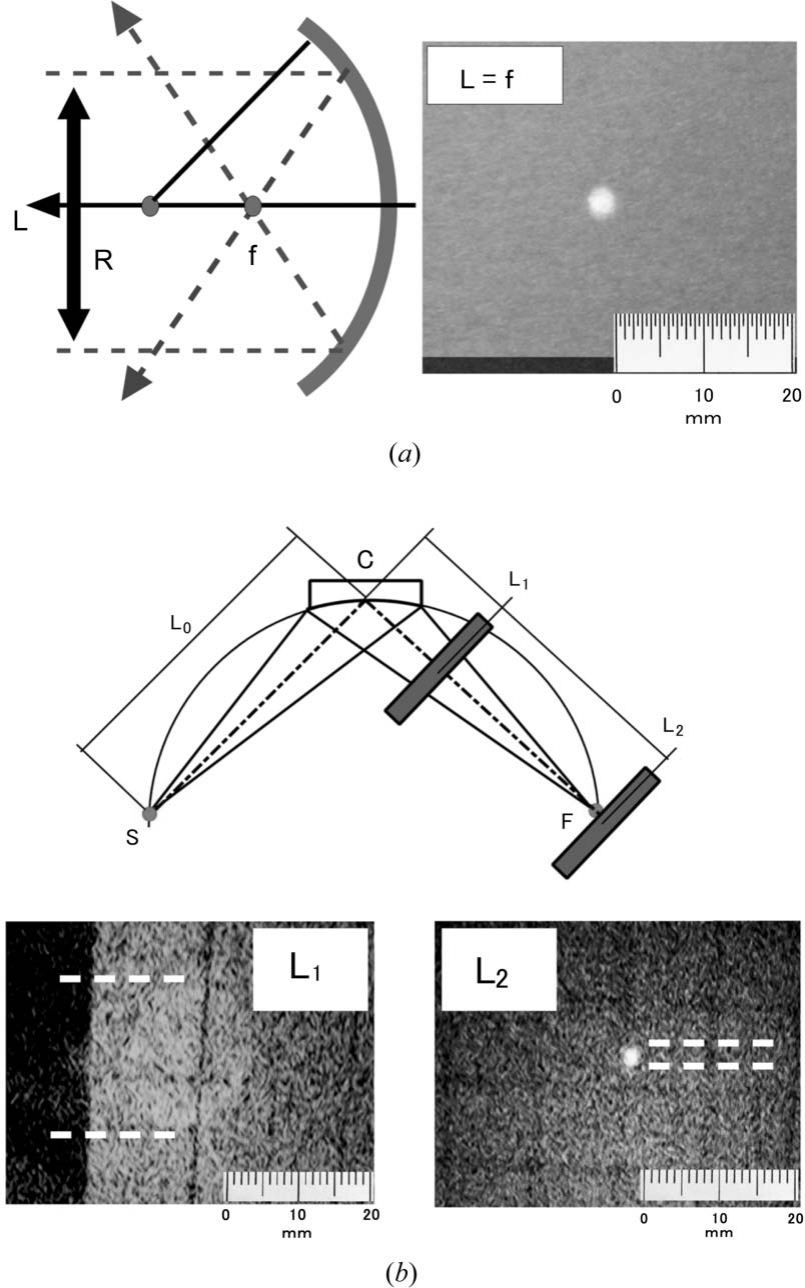
Focusing of (*a*) solar light with a diameter of 20 mm and (*b*) Cu *K*α X-rays by the present crystal. Solar light was focused onto a spot at *f* = 300 mm, showing that the crystal surface was hemispherical with a radius of 600 mm. Cu *K*α radiation was diffracted by the crystal at *C* and focused at *L*2.

Another important condition for realizing a point-focusing monochromator is the curvature of the crystal surface. Under the present diffraction condition the surface must be hemispherical. Since the present process produces a mirror surface, the deformed crystal reflects visible light by acting as a curved mirror. A spherical mirror with a radius of curvature *R* has a focus at *f* = *R*/2, as shown in Fig. 2 ▶(*a*). When solar light with a beam diameter of 20 mm illuminates the present crystal, the image obtained at the focal point, *L* = 300 mm, is found to be a small spot, *i.e.* the surface of the present crystal is hemispherical, as shown in the figure. The spot size measured by full width at half-maximum (FWHM) is 3.0 mm, which agrees with the size of 2.9 mm expected from a convolution of the native divergence of solar light and the beam broadening of the X-ray diffraction discussed below.

Focusing images using Cu *K*α_1_ radiation are shown in Fig. 2 ▶(*b*). An incident X-ray beam generated at a microfocus X-ray generator with a focus size of 70 µm in diameter was shaped by a round slit and projected onto the monochromator crystal as a homogeneous divergent beam with a diameter of about 18 mm. The diffracted beam was imaged on a Gd_2_O_3_S fluorescence screen and recorded by a charge-coupled device (CCD) camera. The source-to-crystal and crystal-to-focus distance was 2^1/2^ *R* = 850 mm for the symmetric 333 diffraction condition of the crystal with *R* = 600 mm. As shown in the upper diagram, the diffraction images are obtained at 60 mm from the crystal (*L*1) and 850 mm from the crystal (*L*2, at the focal point). The image at *L*1 shows that the diffraction condition is satisfied simultaneously over the entire illuminated area. The pattern converges to a small round spot of about 1.7 mm diameter at the focal point, *L*2, demonstrating that the point focus condition is satisfied. The FWHM of the rocking scan in Fig. 1 ▶ is about 0.023 (7)°. A simple calculation for the broadening at the focus due to the FWHM of the rocking curve thus gives 0.7 (2) mm. Therefore, about half of the broadening of the X-ray beam at the focus is explained by the quality of crystal. To realize a better focus size, a more quantitative understanding of hot plastic deformation of single crystalline wafers is necessary for precise deformation.
